# Epigenetics in Myeloproliferative Neoplasms

**DOI:** 10.1111/jcmm.13095

**Published:** 2017-07-04

**Authors:** Suzanne McPherson, Mary Frances McMullin, Ken Mills

**Affiliations:** ^1^ Blood Cancer Research Group Centre for Cancer Research and Cell Biology Queens University Belfast Belfast UK; ^2^ Centre for Medical Education School of Medicine Dentistry and Biomedical Sciences Queens University Belfast Belfast UK

**Keywords:** myeloproliferative neoplasm, epigenetics, cell signalling, janus kinase, DNA methylation, histone modification, miRNA regulation, combination epigenetic therapy

## Abstract

A decade on from the description of *JAK2 V617F*, the MPNs are circumscribed by an increasingly intricate landscape. There is now evidence that they are likely the result of combined genetic dysregulation, with several mutated genes involved in the regulation of epigenetic mechanisms. Epigenetic changes are not due to a change in the DNA sequence but are reversible modifications that dictate the way in which genes may be expressed (or silenced). Among the epigenetic mechanisms, DNA methylation is probably the best described. Currently known MPN‐associated mutations now include *JAK2, MPL, LNK, CBL, CALR, TET2, ASXL1, IDH1, IDH2, IKZF1* and *EZH2*. Enhancing our knowledge about the mutation profile of patients may allow them to be stratified into risk groups which would aid clinical decision making. Ongoing work will answer whether the use of epigenetic therapies as alterative pathway targets in combination with JAK inhibitors may be more effective than single agent treatment.

## Introduction

Genomic instability is fundamental to the development of cancers 1. The myeloproliferative neoplasms (MPNs) arise when acquired mutations in haematopoietic stem cells cause a change from the polyclonal haematopoiesis seen in health to an abnormal monoclonal haematopoiesis 2. The classic *BCR/ABL*‐negative MPNs include polycythaemia vera (PV), essential thrombocythaemia (ET) and primary myelofibrosis (PMF). They are defined, respectively, by the excess production of erythrocytes, platelets and bone marrow fibrosis. The MPN symptom burden is heterogeneous, ranging from patients who are relatively asymptomatic to those suffering with severe constitutional symptoms, thrombotic disease or transformation to secondary acute myeloid leukaemia (AML). Transformation to AML is the most devastating complication experienced by MPN patients, seen in 20% of PMF, 4.5% in PV and 1% in ET 2, 3, 4, 5. It is usually refractory to treatment and in the majority of cases patients ultimately die of their disease. Therefore, early predictors of leukaemic risk are hugely important 6. A single‐point mutation in the tyrosine kinase *JAK2* is present in the majority of patients and has become part of the formal diagnostic criteria for these conditions 7, 8, 9, 10. However, it is not present in every case and does not explain the phenotypic differences between these three diseases and even between patients. A decade on from the description of *JAK2 V617F*, the MPNs are circumscribed by an increasingly intricate landscape 11. The influence of molecular abnormalities beyond the JAK/STAT pathway is ever more being scrutinised. There is now evidence that the MPNs are likely the result of combined genetic dysregulation with several mutated genes involved in the regulation of epigenetic mechanisms. They appear to be the most frequent after *JAK2 V617F* and *CALR* mutations. In this review, we explore the evidence for epigenetic dysregulation in the MPNs, where recent advances may be helpful in the need for risk‐adapted therapeutic stratification.

## Mutations affecting cell signalling

**Figure 1:**
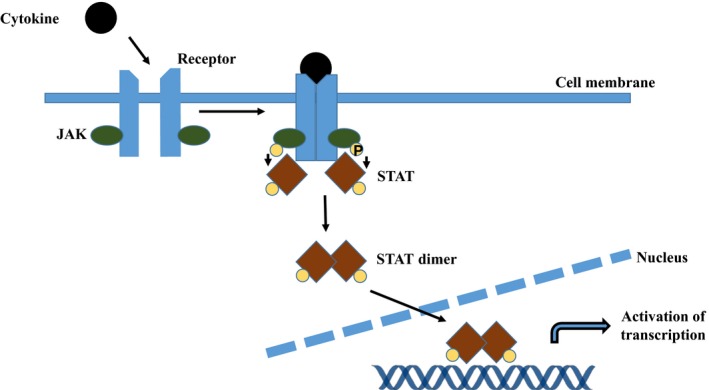
JAK/STAT pathway. A cytokine stimulates the cell surface receptor and activates the associated Janus kinase (JAK). This protein autophosphorylates and binds to a STAT (signal transducer and activator of transcription) protein, which is in turn itself is phosphorylated. The STAT protein then dimerizes and translocates into the nucleus of the cell. Here, it binds to DNA at a promoter site and causes gene transcription.

Normal haematopoiesis is regulated mainly by cytokines such as erythropoietin (EPO), granulocyte colony‐stimulating factor (GCSF) and thrombopoietin (TPO). These cytokines stimulate receptors on the surface of cells and activate the associated Janus kinase (JAK). This protein autophosphorylates and binds to a STAT (signal transducer and activator of transcription) protein, which is in turn itself phosphorylated. The STAT protein then dimerizes and translocates into the nucleus of the cell. Here, it binds to DNA at a promoter site and causes gene transcription (Fig. 1: JAK/STAT pathway). STAT proteins affect basic cell functions such as cell growth, differentiation and apoptosis.

**Figure 2:**
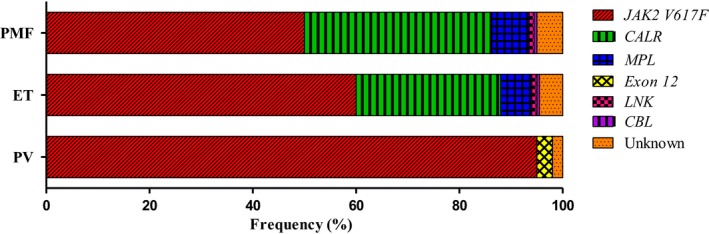
Frequency of MPN‐specific mutations. *JAK2 V617F* Janus kinase 2, valine to phenylalanine at codon 617. *CALR* Calreticulin. *MPL* Myeloproliferative leukaemia virus oncogene. *Exon 12* Janus kinase exon 12. *LNK* Lymphocyte‐specific adaptor protein. *CBL* Casitas B cell lymphoma.

The point mutation *JAK2 V617F* was initially described in 2005 2, 12, 13, 14, 15, located in exon 14 of the gene, and it results in a single base change from guanine to thymine at nucleotide position 1849, leading to an amino acid change from valine to phenylalanine at codon 617. This leads to constitutively active JAK2 kinase signalling that is independent of cytokine stimuli. Approximately 95% PV patients, 50–70% ET patients and 40–50% PMF patients possess the JAK mutation 2, 16. However, initial excitement was short‐lived when it became clear that its role in MPNs was not as pathognomonic as *BCR/ABL* in chronic myeloid leukaemia (CML) 1. *JAK2 V617F* positivity or high mutant allele burden does not seem to have a clear association with survival, leukaemia transformation or risk of thrombosis 17. In recent years, several other mutations in this pathway have been described. A second mutation in *JAK2* can occur at exon 12 with similar functional consequences to the canonical mutation 2, 18. It occurs in a small percentage of *JAK2 V617F*‐negative PV patients, but not in ET or PMF. The myeloproliferative leukaemia virus oncogene (*MPL*) encodes the receptor for TPO *via* JAK‐STAT signalling. Somatic mutations affecting exon 10 of MPL are seen in up to 15% of *JAK2 V617F*‐negative ET and MF patients 2, 19, 20. This gain‐of‐function mutation is due to a substitution of tryptophan to leucine at codon 515 and leads to constitutive phosphorylation of JAK2, STAT3, STAT5, ERK and AKT proteins 21, 22, 23. Lymphocyte‐specific adaptor protein (LNK) negatively regulates TPO/EPO signalling, and a loss‐of‐function mutation is found here at low frequency in JAK2‐negative patients 24, 25. Further downstream, mutations in suppressors of cytokine signalling (*SOCS*) and Casitas B cell lymphoma (*CBL*) have been seen rarely in within MPN patients. In 2013, a mutation in exon 9 of the calreticulin (*CALR*) gene was identified, by next‐generation sequencing (NGS), in approximately 73% of *JAK2/MPL*‐negative ET and MF patients 26, 27. *CALR* is present in the endoplasmic reticulum, where it forms a key component of the quality‐control machinery that ensures proper glycoprotein folding and it also contributes to calcium homeostasis. Recent work has demonstrated the exact mechanism by which *CALR* causes the MPN phenotype, where the mutant proteins interact with the thrombopoietin receptor (MPL) and directly lead to dimerization and activation of JAK2 28, 29, 30. Retrospective analysis of clinical data suggests that *CALR*‐positive patients have a more benign clinical course than patients with the same MPN who are *JAK2* or *MPL* positive 26. Compared to *JAK2* patients, *CALR* patients have a lower risk of thrombosis and longer overall survival (OS) 26. Identification of these additional mutations means that up to 95% of patients can now be reliably diagnosed (Fig. 2: Frequency of MPN‐specific mutations).

## Mutations in epigenetic‐associated genes

In a study using targeted NGS of 104 cancer‐related genes on 197 MPN patients to examine clonal evolution over time 31, approximately 10% of patients had no mutation detectable in any of the genes analysed and 54% had mutations only in *JAK2 V617F* or *CALR*. These patients were found to have the most favourable prognosis and the lowest risk of disease progression. The remaining 36% had additional mutations detected, other than *JAK2 V617F* or *CALR*. Most of these were mutations affecting genes implicated in epigenetic regulation (*TET2* 12%, *ASXL1* 5%, *DNMT3a* 5%, *EZH2* ~3% and *IDH1* ~1.5%) 31. Many mutations in epigenetic‐associated genes are not exclusive to MPN and are seen in a wide spectrum of myeloid malignancies. They can also coexist with *JAK2/CALR/MPL* suggesting that in some cases, these two classes of mutations may combine together produce the individual MPN phenotype. It is thought that the rate of clonal evolution in MPN is slow and most mutations are already present at diagnosis. However, the order in which mutations are acquired may also affect disease phenotype. Of note, the reversible nature of epigenetic changes may make them good potential therapeutic targets.

Epigenetic changes are not due to a change in the DNA sequence but are reversible modifications that dictate the way in which genes may be expressed (or silenced). It can do this through alterations in DNA methylation, modification of histones or chromatin structure, or *via* changes to RNA.

**Figure 3:**
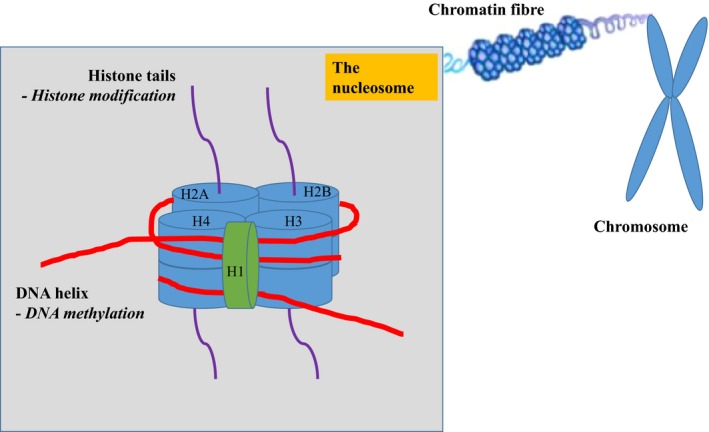
The nucleosome. This structure consists of a core of eight histone proteins (two of histone H2A, H2B, H3 and H4) around which DNA is tightly coiled. It is further stabilized histone H1.

To understand these epigenetic changes, it is essential to understand the structures that aid the packaging of DNA within the cell nucleus (Fig. 3: The nucleosome). The nucleosome consists of a core of eight histone proteins (two of histone H2A, H2B, H3 and H4) around which DNA is tightly coiled. This structure is further stabilized by histone H1. DNA is negatively charged, while lysine residues on histones hold a positive charge. The nucleosome is not static and changes in the condensed nature of chromatin signal for changes in gene transcription.

## JAK2

**Figure 4:**
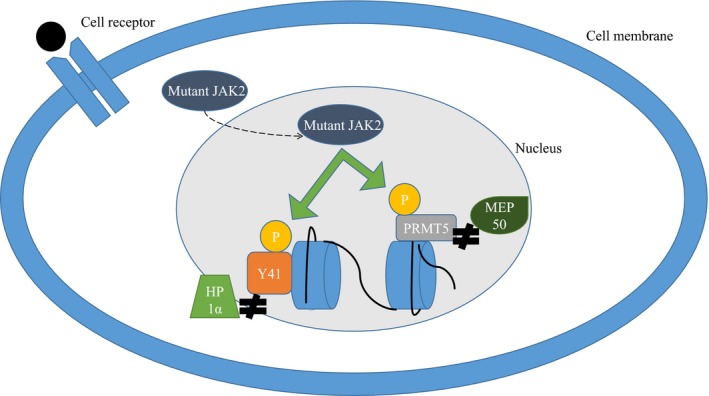
The epigenetic effects of JAK2. Mutant JAK2 may have an epigenetic role through its presence in the nucleus where it can lead to phosphorylation of histone H3 and the arginine methyltransferase PRMT5. Phosphorylation of H3 at position Y41 blocks the binding of heterochromatin protein 1α (HP1α), and phosphorylation of PRMT5 prevents its association with methylosome protein 50 (MEP50).

In addition to its prominent role in cytokine signalling, mutant JAK2 may have an epigenetic role through its presence in the nucleus where it can lead to phosphorylation of histone H3 and the arginine methyltransferase PRMT5 32, 33 (Fig. 4: The epigenetic effects of *JAK2*). Phosphorylation of H3 at position Y41 blocks the binding of heterochromatin protein 1α leading to changes in gene transcription, DNA repair and other cellular processes 21. Phosphorylation of PRMT5 prevents its association with methylosome protein 50 (MEP50), leading to decreased methylation of histones H2A and H4.

## DNA methylation

**Figure 5:**
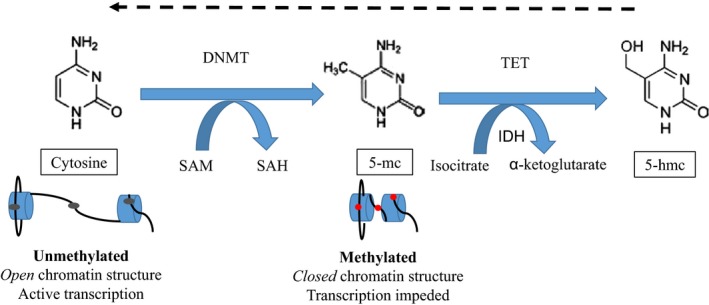
Overview of DNA methylation. Cytosines are methylated by the addition of a methyl group to the 5 carbon ring of the pyrimidine ring under the action of DNA methyltransferase (DNMT) enzymes; the 5‐methylcytosine (5‐mc) formed acts to repress gene transcription. The methyl group (CH_3_) is donated by S‐adenosyl methionine (SAM), which itself is reduced to S‐adenosyl homocysteine (SAH). Conversely, the ten‐eleven translocation (TET) proteins belong to a family of alpha‐oxaloglutarate dependent enzymes which catalyse the conversion of 5‐mc to 5‐hydroxymethylcytosine (5‐hmc), an initial step in demethylating DNA that ultimately leads back to an upregulation of transcription. Isocitrate dehydrogenase (IDH) enzymes catalyse the conversion of isocitrate to α‐ketoglutarate, a reaction that is required for the function of TET enzymes.

Among the epigenetic mechanisms, DNA methylation is probably the best described. The addition of methyl groups to DNA typically occurs predominately at CpG sites (cytosine and guanine separated by one phosphate in the linear sequence of bases along the length of the DNA) prior to the promoter regions of genes 34, 35. Cytosines are methylated by the addition of a methyl group to the five carbon ring of the pyrimidine ring under the action of DNA methyltransferase (DNMT) enzymes; the 5‐methylcytosine (5‐mc) formed acts to repress gene transcription. The methyl group (CH_3_) is donated by S‐adenosyl methionine (SAM), which itself is reduced to S‐adenosyl homocysteine (SAH). Conversely, the ten‐eleven translocation (TET) proteins belong to a family of alpha‐oxaloglutarate‐dependent enzymes which catalyse the conversion of 5‐mc to 5‐hydroxymethylcytosine (5‐hmc), an initial step in demethylating DNA that ultimately leads back to an upregulation of transcription. Isocitrate dehydrogenase (IDH) enzymes catalyse the conversion of isocitrate to α‐ketoglutarate, a reaction that is required for the function of TET enzymes. These mechanisms (Fig. 5: Overview of DNA methylation) help to regulate gene expression in normal development. In the development of cancer, a gain of methylation in a tumour suppressor gene would lead to gene inactivation and a loss of methylation in proto‐oncogenic genes would lead to gene expression 36.

Mutations of *TET2* impair the function of TET enzymes, resulting in reduced levels of 5‐hmc. As discussed above, they are seen in 16% PV, 5% ET and 17% PMF 37. They also occur in mastocytosis, AML, myelodysplastic syndromes (MDS) and more than half of chronic myelomonocytic leukaemia (CMML) cases 37, 38. Murine models with TET2 mutations had an expansion of the haematopoietic stem cell compartment and in particular demonstrated myelomonocytic proliferation 39. In one study of MPN patients by Lundberg *et al*., *TET2* mutations were seen to confer a high risk of leukaemic transformation (increased by 30%) and shorter OS 31. In addition, there is evidence that *TET2* mutations appear to be an early event that may provide clonal advantage and set up a ‘fertile ground’ for MPN disease initiation 40, 41.

DNMT 3A and 3B (alpha and beta) carry out *de novo* methylation while DNTM1 maintains existing methylation patterns after cell division. Mutations in DNMT3A were initially found in AML patients and confer an intermediate to adverse prognosis. They have been seen less frequently in MPN, in approximately 10% of patients 2. They may cause a gain or loss of function. The most common mutation R882H causes a loss of function and the consequently reduced methylation acts to increase gene transcription with murine models displaying myeloid proliferation with thrombocytosis. Initially, DNMT3A mutations were only reported early in myeloid disease, prior to gaining *JAK2* 42. However, it seems that these mutations can also be acquired late in disease development and the order of acquisition may be associated with differences in MPN phenotype 42. When they occur prior to *JAK2,* they are associated with ET, whereas the reverse is associated with PV. However, studies to date have not clearly linked *DNMT* mutations with clinical outcome in MPN patients.

The enzymes IDH 1 and 2 (*IDH1/2*) map to chromosomes 2 and 15, respectively. *IDH1/2* mutations were first described in 2008 in gliomas and occur at low frequency in MPN. The largest study of IDH mutations in MPN reported frequencies of approximately 2% in PV, 1% ET, 4% PMF and 22% in blast phase MPN 43. In blast phase, MPN *IDH* mutational status predicted poor prognosis.

Most DNA methylation studies in MPN have been carried out looking at single‐gene mutations, but one study has looked at the epigenetic landscape by examining global DNA methylation in 71 chronic PV/ET/PMF patients 36. An aberrant methylation pattern was seen in these patients when compared to healthy controls, but no difference was seen between the three MPN phenotypes. However, in 13 of patients samples taken during transformation to secondary AML were also examined and these had an increased number of differentially methylated regions when compared to the samples from chronic patients 36. Interestingly, analysis of the enriched genes indicated they were involved in important signal transduction pathways such as NFkB (nuclear factor kappa light‐chain enhancer of activated B cells) and binding sites for important transcription pathways such as globin transcription factor 1 (GATA1). GATA1 has critical functions in erythrocyte development including establishment of the cytoskeleton and polypeptide globin chains. In patients who transformed to AML, genes in the interferon (IFN) pathway were seen to be hypermethylated, which may be important given IFN has a role in tumour surveillance.

## Histone modifications and regulation of chromatin structure

The N terminal lysine residues on histones are prone to post translational modification, leading to transcriptional activation or repression. These covalent modifications can include acetylation, methylation and phosphorylation, among others. Acetylation occurs at lysine (K) residues particularly on histone H3 and H4. Histone acetyl transferases are enzymes that aid the transfer of acetyl groups, while histone deacetylases (HDACs) remove acetyl residues from histone tails. Disruption of this equilibrium can play a role in the development of cancer and have been described in AML. The methylation of histones takes place on lysine, arginine and histidine residues. An additional layer of complexity is added as lysines may be mono‐ (me), di‐ (me2) or tri‐ (me3) methylated. As with DNA, SAM donates the methyl group under the action of histone methyltransferase (HMT) enzymes. Certain commonly studied histone methylation marks are in general associated with transcription activation (H3K4: histone H3 at lysine (K) amino acid 4) while others are associated with transcriptional repression (H3K9).

Yet little is known about the role of individual histone modifications within MPNs, and current research has focused mainly on changes relating to the polycomb group proteins. These are a family of proteins which can remodel chromatin thus exerting a repressive effect on gene expression. Polycomb repressive complex 2 (PRC2) catalyses di‐ and trimethylation of lysine 27 on histone H3 (H3K27me2/3), leading to gene silencing. Addition of sex combs like protein 1 (*ASXL1*) maps to chromosome 20 and is thought to be an important mediator of PRC2 function 44. Mutations in *ASXL1* were first described in 2009 in MDS/AML 45. Studies of the effect of *ASXL1* mutations in MDS patients demonstrated that they predicted for worse outcome 46. In murine models, loss of *asxl1* resulted in progressive multilineage cytopenias and dysplasia 47. Deletions, insertions and nonsense mutations of ASXL1 have been found in 3% ET and 13% PMF 37. Among patients with MPNs, ASXL1 mutations appear to be commoner in older patients as well as in patients with PMF or post‐ET/PV MF. In PMF, they are associated with a more severe anaemia and the inferior survival of patients 48.

*EZH2* (enhancer of zeste homolog 2) is another key regulator of PRC2 49. *EZH2* is present on chromosome 7 and is overexpressed in solid tumours such as lymphoma. MPN‐associated EZH2 mutations result in a loss of function and may have tumour suppressor activity 37. Both *EZH2* and *ASXL1* are thought to occur later in MPN development, but to date, there is no definite evidence that they carry any independent prognostic value.

## miRNA regulation

Changes in the regulation of RNA can lead to cell cycle arrest and apoptosis. Described mutations here include *SF3B1, SRSF2* and *IKZF1* (IKAROS family zinc finger 1). *SF3B1* mutations are seen most often in a subtype of MDS (RARS—refractory anaemia with ring sideroblasts), while *SRSF2* is seen in CMML. They can also occur at low frequency in MPN patients (around 5%) with the suggestion that *SRSF2* confers poor prognosis in MF. *IKZF1* deletions are rare in chronic phase MPN but have been detected at transformation to AML. They have been previously described mainly in lymphoid diseases and are believed to modulate expression of lineage‐specific genes 17.

## JAK inhibition and potential for combination with epigenetic therapies

Disease complications are a leading cause of morbidity and mortality in MPN patients and play an important role in determining when to initiate treatment 40. Current prognostic scoring systems for ET and PV do not take into account any genetic markers and are based solely on patient age, blood counts and development of complications. Standard treatments for the classic *BCR/ABL*‐negative MPNs include low‐dose aspirin, cytotoxic drugs such as hydroxycarbamide or anagrelide, venesection and IFN. In younger patients with high‐risk MF or transformation to secondary AML, intensive chemotherapy followed by bone marrow transplantation may be considered. In 2011, the JAK1/2 inhibitor ruxolitinib (also known as INCB18424) was approved for the treatment of intermediate‐risk and high‐risk myelofibrosis. In clinical trials, when it was compared to the best available therapy (BAT), ruxolitinib resulted in a rapid reduction in spleen size (35% or more from baseline by MRI or CT) and also improved constitutional symptoms (including anorexia, shortness of breath, fatigue, insomnia and pain) leading to improved quality of life 50. In a subsequent 3‐year follow‐up study (COMFORT‐II), longer overall survival (OS) was also seen in patients on continuous ruxolitinib 51. This medication is now being trialled in PV and ET. ‘RESPONSE’ evaluated the safety and efficacy of ruxolitinib in 110 PV patients, compared to BAT. Ruxolitinib was superior in controlling haematocrit, reducing spleen volume and improving symptoms such as sweats, itch, lethargy and abdominal discomfort 52. Preliminary reports from the investigator lead study ‘MAJIC’ suggest that for the treatment of ET, ruxolitinib offered no advantage in terms of complete haematological remission rates, compared to BAT. However, once again benefits were seen for constitutional symptoms with patients reporting improvement in pruritus, weight loss and early satiety 53. Despite clear clinical benefits, treatment with ruxolitinib does not seem to reduce mutant allele burden, neither in patients nor in mouse models 54. This is in contrast to the effect of the tyrosine kinase inhibitors on *BCR/ABL* in CML and has caused researchers to question whether *JAK2* is an essential therapeutic target in MPN. The concept of targeted therapy in MPN may be more complicated than first thought. Other JAK inhibitors are in various stages of investigation for the treatment of MF. These include CYT387 which has demonstrated efficacy in reducing spleen size as ruxolitinib did, but with the added benefit of decreasing anaemia 55. Pacritinib (SB1518), a dual JAK2/Fms like tyrosine kinase 3 inhibitor, has also proved efficacious in reducing spleen size and constitutional symptoms and is being trialled in patients with low baseline platelet counts (PERSIST‐1 and PERSIST‐2) 56, 57. A major advantage of these trials against the ruxolitinib trials was that patients with any degree of cytopenias were eligible. However, the US Food and Drug Administration (FDA) placed a full clinical hold on pacritinib following reports of patient deaths related to cardiac failure and intracranial haemorrhage. The full clinical data from these studies are under review, and further dose finding studies may have to be performed. Several other agents including fedratinib (SAR302503), CEP‐701, XL019, LY278544, BMS‐911543 and AZD 1480 have been removed from trials due to various complications including neurological toxicities, myelosuppression or insufficient efficacy 40.

A variety of novel small molecule therapies are under investigation in combination with JAK inhibitors. Given the landscape of epigenetic mutations alongside JAK2 mutations, it is possible that drugs such as histone deacetylase inhibitors (HDAC) like Givinostat 58, 59, telomerase inhibitors (imetelstat), or DNMT inhibitors could work synergistically with JAK inhibitors. There is no question that hypomethylating agents (azacytidine and decitabine) have offered clinical benefit to selected MDS/AML patients 60. Other targeted therapies include PI3K (phosphoinositide 3‐kinase) inhibitors or mammalian target of rapamycin (mTOR) inhibitors which target enzymes which are part of the PI3K/AKT/mTOR pathway 40. There are many components within this pathway and inhibition may result in tumour suppression. A trial of a PI3K inhibitor used in combination with ruxolitinib showed antiproliferative effects 61. Despite recent advances, the true role of molecular status in the choice of treatment of individual patients requires further work before it can be applied routinely 40.

## Conclusions

Mutations in MPN are involved in a wide range of cellular pathways including cytokine receptor signalling but also the regulation of gene expression 2. Currently known MPN‐associated mutations include *JAK2, MPL, LNK, CBL, CALR, TET2, ASXL1, IDH1, IDH2, IKZF1* and *EZH2* (Table 1: Mutations in epigenetic regulators and their current prognostic value in myeloid malignancies) 36. In other myeloid malignancies, AML and myelodysplastic syndrome (MDS), the number of mutations at diagnosis correlates with time to leukaemic transformation. In these patients, molecularly guided therapeutic trials have led to improved risk stratification and the development of targeted therapy 6. However, in MPN, certain mutations may be present at disease initiation and others acquired in a random fashion throughout the disease course. In addition, some of these mutations are not mutually exclusive, making the hierarchy complex and unpredictable. This makes it more difficult to predict MPN patient outcomes at a single time‐point 6. More than a decade on from the original description of *JAK2 V617F*, it seems that contributing the MPN phenotype to constitutive activation of JAK2 is to over simplify the complex molecular interactions that regulate the JAK/STAT pathway. Along with the recent description of the *CALR* mutation, differences in the epigenetic landscape have been hypothesised to be a key component, with studies showing aberrantly methylated genes in MPN patients particularly at transformation to AML 36. Other important insights into MPN pathogenesis have appeared with TET2 seeming to be an important driver of leukaemic transformation 62. Enhancing our knowledge about the mutation profile of patients may allow them to be stratified into risk groups which would aid clinical decision making. Management of MPN patients has now evolved and requires carefully consideration of individual patient disease burden, comorbidities and molecular status. JAK inhibitors have had a meaningful impact as single agent therapy in PMF and now potentially resistant cases of PV and ET. Ongoing work will answer whether the use of epigenetic therapies as alterative pathways in combination with JAK inhibitors may be more effective than single agent treatment.

**Table 1:** Mutations in epigenetic‐associated genes and their current prognostic value in myeloid malignancies

Mutation	Area of regulation	MPN effect	Significance in MDS/AML
*JAK2*	Histone modification	Unknown	No data, rare event in de novo AML
*TET2*	DNA methylation	Poor risk, seen at leukaemic transformation	No clear significance in MDS Worse prognosis in normal karyotype AML
*DNMT3A*	DNA methylation	Unknown	Adverse prognosis
*IDH1/2*	DNA methylation	Poor risk at blast phase	No clear importance
*ASXL1*	Histone modification	Poor risk	Poor risk
*EZH2*	Histone modification	Unknown	Adverse outcome in CMML and AML
*SF3B1*	miRNA regulation	Unknown	Good prognosis, seen commonly in MDS‐RS
*SRSF2*	miRNA regulation	Poor risk	Worse prognosis, clustered in MDS with excess blasts
*IKZF1*	miRNA regulation	Poor risk	Unclear, possible link with AML Monosomy 7

*JAK2*, Janus kinase 2; *TET2*, Ten‐eleven translocation 2; *DNMT3A*, DNA methyltransferase 3A; *IDH1/2*, Dehydrogenase 1/2; *ASXL1*, Addition of sex combs like protein 1; *EZH2*, Enhancer of zeste homolog 2; *SF3B1*, Splicing factor 3 subunit 1; *SRSF2*, Serine and arginine splicing factor 2; IFZF1, IKAROS family zinc finger 1; RS, Ring sideroblasts.

## Conflict of interest

The authors confirm that there are no conflicts of interest.
